# Spatial variation in the littoral vertebrate community of a reservoir relative to physical and biological gradients

**DOI:** 10.7717/peerj.693

**Published:** 2014-12-11

**Authors:** Nathan Ruhl, Jessica J. Soski, Willem M. Roosenburg

**Affiliations:** 1 Biological Sciences Department, Ohio University, Athens, OH, USA; 2 Biological Sciences Department, Rowan University, Glassboro, NJ, USA

**Keywords:** Reservoir, Fish, Turtle, Condition, Resource, Gradient, NMDS

## Abstract

Reservoirs possess gradients in conditions and resources along their long (deep-shallow) axis, but the response of littoral vertebrates (fish and turtles) to these gradients is poorly understood. We have quantified the littoral vertebrate communities throughout a small reservoir in Southeastern Ohio during July and August using traps, and related community composition to environmental variables using NMDS ordination. Ordination revealed that fish and turtles were broadly separated in ordination space, and three distinctly different environmental gradients were significantly associated with the underlying observed species abundances. Observed turtle abundance was explained by measurements of bathymetry, turbidity, and benthic resources, but none of these environmental variables were a reliable predictor of observed fish abundance. Temperature was a poor predictor of observed abundance for both fish and turtles independently, but when fish and turtles were considered together, it became apparent that there were cold areas of the reservoir where observed fish and turtle abundances were different than in other areas of the reservoir. These results suggest that the predictor (environmental) variables we used were appropriate for investigating turtle ecology in reservoirs, but that observed fish abundance is mediated by factors that were not modeled. The efficacy of using traps, the ecological implications of considering fish and turtles together as sympatric and potentially competing species, and directions for future study are discussed.

## Introduction

Many studies have investigated the effect of environmental variation on differences between fish populations (e.g., Carey & Mather, 2009) and communities (e.g., Tonn & Magnuson, 1982; Mehner et al., 2005) among lakes. These studies typically treat lakes as homogenous with respect to environmental conditions and attempt to relate population or community dynamics to representative environmental variables. This same approach has been used to distinguish fish assemblages of reservoirs as a function of the environmental conditions in the lacustrine zone (e.g., Godinho, Ferreira & Castro, 1998; Irz et al., 2002; Hoxmeier, Aday & Wahl, 2009). While this approach works well in lakes, it may be problematic in reservoirs, because most reservoirs exhibit distinct gradients in a suite of environmental variables and resource availability along their horizontal (longitudinal) axis (e.g., dissolved solids, Atkinson & Mabe, 2006; turbidity, Thornton, 1990a; littoral zone slope, Thornton, 1990a; temperature, Ford, 1990; nutrient availability, Kennedy & Walker, 1990; phytoplankton, Caputo et al., 2008).

**Figure 1:**
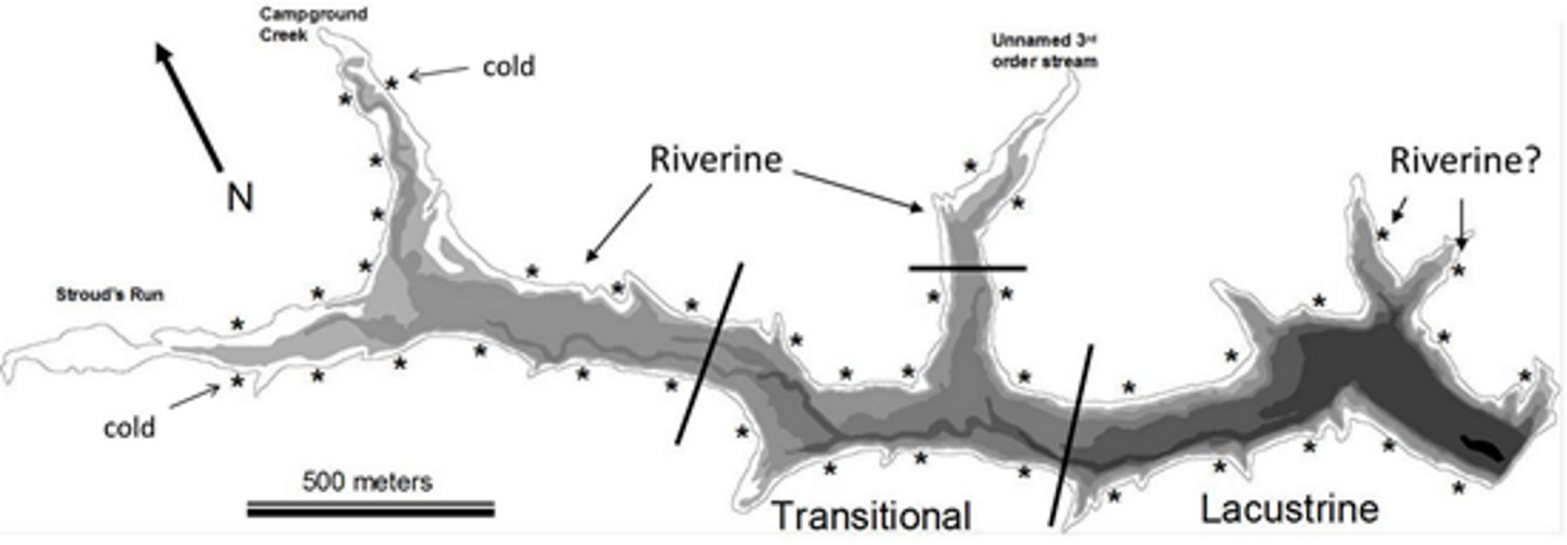
Bathymetric map of Dow Lake. Asterisks indicate the approximate position of a trapping site, bathymetric layers occurs every 1.5 m, and black lines delineate the extent of the different reservoir zones used in Figs. 3, 4 and 6. “Riverine?” indicates the position of two “Lacustrine” sites found to be similar in community composition to riverine sites in the NMDS ordination (Fig. 4). The “cold” tag indicates two sites that were characterized by distinctly lower temperature (Figs. 6C and 7).

The presence of environmental and resource gradients in reservoirs has led to a simple classification scheme of functionally different areas within a reservoir (Kimmel & Groeger, 1984): the riverine zone is the shallow, well-mixed portion, the lacustrine zone is the stably stratified lake-like area, and the transitional zone is the area of mixed dynamics that separates the other two zones (Fig. 1). This zonation scheme has not been widely adopted by researchers studying vertebrates (fishes and turtles), but is often used in the context of understanding reservoir plankton dynamics (Bernot et al., 2004; Wang et al., 2011).

Studies of fish response to environmental and resource gradients within reservoirs are rare and focus primarily on fish in open water. For example, Prchalová et al. (2008) found that both juveniles and adults inhabiting the limnetic and benthic zones of Želivka reservoir (Czech Republic) increased in abundance with distance from the dam. Similarly, Prchalová et al. (2009) and Vašek et al. (2004) found that most species inhabiting the Rimov Reservoir (Czech Republic), which has never been stocked with lentic species, increased in abundance and biomass with distance from the dam. In the former, the differences were attributed to habitat availability whereas in the latter the differences were attributed to the species composition being poorly adapted for a lentic environment. Studies of fish inhabiting the littoral zone of reservoirs (and lakes) are rare because of the difficulty inherent in accurately sampling these habitats and because of the proportional area of the littoral zone relative to other habitats in natural lakes (Winfield, 2004). With regard to accurate sampling, rigorously quantitative methods used in limnetic habitat (e.g., trawling or hydroacoustics) are not appropriate for littoral habitat and alternatives such as trapping, gill netting, seining, or electroshocking each have biases that need to be recognized (Winfield, 2004; see Discussion). Much of the work investigating the littoral fish community of lakes and reservoirs has focused on ontogenetic habitat shifts (Werner & Hall, 1988; Brosse, Grossman & Lek, 2007) and on shoreline heterogeneity (patchiness) as a driver of community composition/complexity (reviewed by Matthews, 1998).

While some attention has been given to fish inhabiting the littoral zone of reservoirs, very little work has explicitly investigated the role of turtles in relation to reservoir limnology, despite many studies of turtles having been conducted in reservoirs. In one study, Aresco (2009) explicitly investigated the response of the turtle community to limnological variables in Florida lakes and was able to find a number of correlates to community composition; concluding that both predation and competition (intra- and interspecific) influenced community composition. While this study was conducted among lakes, it may provide insight to the role of turtles in a reservoir, where limnology is dynamic. In another study, Ruhl (2013) found that eastern musk turtles (*Sternotherus odoratus*) inhabiting the littoral zone were both larger and encountered more frequently in the riverine portion of a reservoir in Southeastern Ohio (Lake Hope) during the summer months. The fact that we know so little about the role of turtles in reservoirs is problematic because, while turtles are not common in well-understood Northern lakes, they are more common in the mid-latitudes where reservoirs are primarily found (Thornton, 1990b), and may therefore be an important part of the aquatic community (Aresco & James, 2005).

In the study presented here, we investigate how the littoral vertebrate community (fish and turtles) is structured at the species level and along a number of environmental and resource gradients within a reservoir. There are three explicit goals of this study: (1) describe the observed aquatic vertebrate community inhabiting the littoral zone of a reservoir (2) establish environmental variables associated with shifts in observed community composition and (3) assess the appropriateness of the categorical zonation scheme of reservoirs for fish and turtles.

## Methods

### Study site

**Figure 2:**
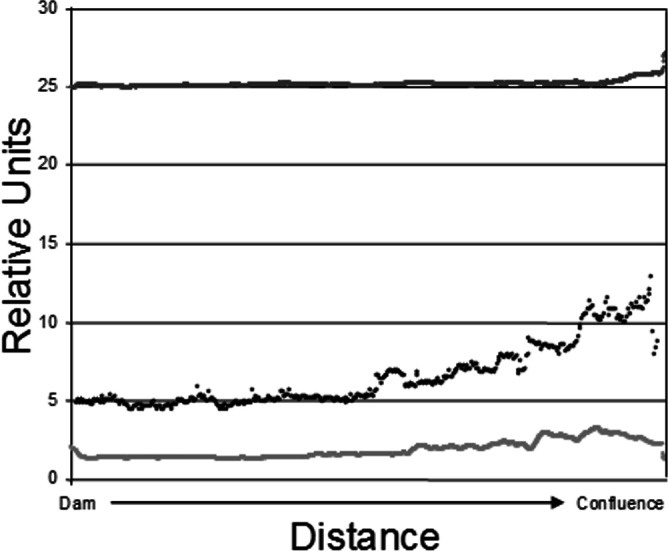
Epilimnetic variation in temperature (dark gray; Celsius), turbidity (black; NTU), and Chl-a concentration (light gray; µg/L) along the long axis of Dow Lake midway through the study (25 July 2007). Note that these data are from the limnetic zone, not the littoral zone where trapping occurred.

Dow Lake is a small 65 hectare reservoir located within the boundaries of Stroud’s Run State Park in the Appalachian foothills of Southeastern Ohio (Fig. 1). Runoff enters the reservoir primarily from Stroud’s Run and Campground Creek (both 3rd order streams) at the northwest end, but a number of smaller watersheds, including an unnamed 3rd order stream entering mid-reservoir, also contribute runoff. Development in the watershed is limited largely to the Stroud’s Run drainage where a small number of houses lie along the river valley. The other watersheds drain moderately sloped and forested hills with minimal development such as campsites and trails. Due to the asymmetry in the source of runoff (the confluence of Stroud’s Run and Campground Creek) and the relatively linear morphology of Dow Lake, environmental gradients from the shallow to deep ends are pronounced (Fig. 2).

### Trapping

We established forty trapping sites approximately equidistant from one another along the shoreline (littoral zone; Fig. 1). The Northeastern shore has a boat launch, swimming beach, and picnic area that could not be trapped due to the risk of equipment theft. We sampled eight sites each week over the course of the 5-week study (July–August 2007). A blocked sampling design ensured that sites throughout the reservoir were sampled each week and that adjacent sites were not trapped in a given week. At each site, two collapsible oval traps (one Promar “large” 81 × 50 × 30 cm, 1 cm mesh size, 12 cm minimum tunnel diameter and one “extra-large” 91 × 62 × 50 cm, 2.5 cm mesh and 15 cm tunnel diameter) were positioned such that they were at least 2 m apart, entrance funnels were parallel with the shoreline, and at a depth that allowed turtles access to air. Therefore, the large traps were positioned at a depth of about 28 cm depth, while the extra-large traps were positioned at a depth of about 48 cm. Each trap was baited with a small amount of commercially available dip bait (Premo brand “original super sticky dip bait”) hung inside the trap in a cheesecloth bag. These traps were then checked and re-baited every 24 h for five days. This study was conducted in accordance with Ohio University’s IACUC guidelines and Ohio Department of Natural Resources #464 sampling permit.

### Analysis

Multidimensional Scaling (MDS), as it relates to this study, is an ordination technique that allows sites and species to be plotted in ordination space based on catch frequency. The goal of MDS is to organize sites in a low number of dimensions (usually 2–3) such that “stress” between objects (sites and species) is minimized. Stress is computed as a function of residuals and is therefore analogous to the “goodness of fit” metric used in ANOVA. When attempting to recover underlying environmental gradients, MDS is generally superior to Principal Components Analysis (PCA), Correspondence Analysis (CA), and De-trended Correspondence Analysis (DCA) because it is more robust against the effects of curvilinear distortion, especially in two dimensions (Minchin, 1987).

Non-metric multidimensional scaling (NMDS) uses rank-order distance rather than the actual distance metric used in MDS. Therefore, the calculated distance between sites (based on species observed abundances) is less important than the rank distance of a site, resulting in an ordination of site objects that better reflects differences in common species and is less influenced by rare species. Because the impact of rare species is moderated by NMDS ordination, the final position of rare species objects is less reliable than in other techniques, but still provides a reasonable representation of the underlying ordering of common and rare objects (species). The NMDS analysis presented here was conducted in R using the vegdist function of the vegan package to calculate a Bray-Curtis dissimilarity matrix and the metaMDS function of the MASS package to actually perform the ordination (Oksanen, 2007). Normally, NMDS must be performed iteratively in order to find the solution (plot of sites and species objects in ordination space) that minimizes disparities between actual dissimilarities and rank-order dissimilarities, but the metaMDS function automatically performs this iterative search for the optimal solution with the best stability.

### Environmental fitting

Environmental vectors were fitted to the ordination using the envfit function of the vegan package in R. Each vector (environmental variable) was independently fitted to the underlying ordination and therefore has no influence in the analysis on the position of species objects, site objects, or other vectors. Because the modeled vectors represent gradients in both highly variable and relatively static conditions and resources, some variables were measured at each site on the same day during trapping, while other variables were measured on the same day at all sites at the end of the study. The fit (*R*^2^) of each variable to the ordination using the envfit function was assessed with a Monte-Carlo analysis of 10,000 permutations.

Turbidity (NTU), *in-vivo* chlorophyll-a (µg/L), and temperature (C) were measured on the same day at all sites being trapped in a given week using an Aquafluor 8,000 handheld fluorometer (Turner Designs, Sunnyvale, CA, USA). The abundance of woody debris, filamentous algae, submerged macrophytes, emergent macrophytes, riparian vegetation, and leaf litter at each site was ranked on a scale of 1–10. Each of these ranks was independent of one another, meaning a structurally simple site could have a rank below 10 and a structurally complex site could have a rank above 10. This is in contrast to the variables representing the substrate, which were ranked on a 1–10 percentage scale where the sum of the four ranks always equaled 10. “Fine” was any material <0.3 cm diameter, “coarse” was 0.3–5.0 cm diameter, “boulder” was >5.0 cm but not embedded, and “bedrock” was large, embedded rock. Littoral zone slope was expressed as depth (cm) at 3 m distance perpendicular to shore at each site. Light intensity was measured at water level using a Solarlight PMA 2100 handheld meter (Solarlight, Glenside, PA, USA).

Maximum depth (Zmax) and average depth (Zmean) were determined by creating a depth profile across the short axis of the reservoir at each site using a line and weight cast from a boat. Because most sites were paired with each other on opposite sides of the reservoir, this profile usually linked two sites, causing the depths to be shared between sites. In cases where sites were not paired (e.g., opposite the developed northeastern shore) profiles were still conducted perpendicular to shore and were unique to those sites. Note that because the process of fitting variables to the NMDS ordination is independent for each variable, and has no influence on the underlying ordination, correlated variables such as maximum and mean depth are allowed. See Table 1 for a tabular summary of these variables.

**Table 1:** Summary of environmental variables fitted to the NMDS ordination with fitting statistics (coefficient of determination and corresponding *p*-value).

Measured	Unit	Variable	Mean	SE	Range	*R* ^2^	*p*
At-trapping	NTU	Turbidity	4.46	0.79	26.81	0.306	0.003
	µg/L	Chlorophyll-a	0.12	0.02	0.8	0.01	0.826
	Celsius	Temperature	28.25	0.15	4.5	0.171	0.029
	Scale (1–10)	Woody debris	1.43	0.2	5	0.011	0.819
	Scale (1–10)	Algae/Macro.	2.45	0.39	9	0.01	0.147
	Scale (1–10)	Emerg. Macro.	0.3	0.17	6	0.035	0.498
	Scale (1–10)	Riparian veg.	3.45	0.18	4	0.049	0.385
	Scale (1–10)	Leaf litter	0.38	0.13	4	0.155	0.042
Post-trapping	Percentage	Bedrock	0.33	0.15	4	0.0159	0.741
		Boulder	1.58	0.28	8	0.156	0.046
		Coarse	2.4	0.36	8	0.079	0.221
		Fine	5.7	0.56	10	0.128	0.088
	Z 3 m offshore	Shoreline slope	70.27	5.56	132.9	0.171	0.033
	Meters	Zmax	5.28	0.52	11.9	0.288	0.001
	Meters	Zmean	3.63	0.32	6.56	0.314	0.001
	Lux	Surface light	907	95	1935	0.04	0.468

The data used to construct Fig. 2 were obtained by slowly towing Hach DS-5 (Hach Company, Loveland, CO, USA) and In-Situ Troll 9000P (In-Situ Inc, Fort Collins, CO, USA) sondes at a depth of 1 m along the long axis of the reservoir. The sampling tract ran from near the dam to the confluence of Stroud’s Run and Campground Creek. Measurements were recorded every two seconds in the case of temperature and chl-a and every five seconds for turbidity.

## Results

### Species encountered

**Figure 3:**
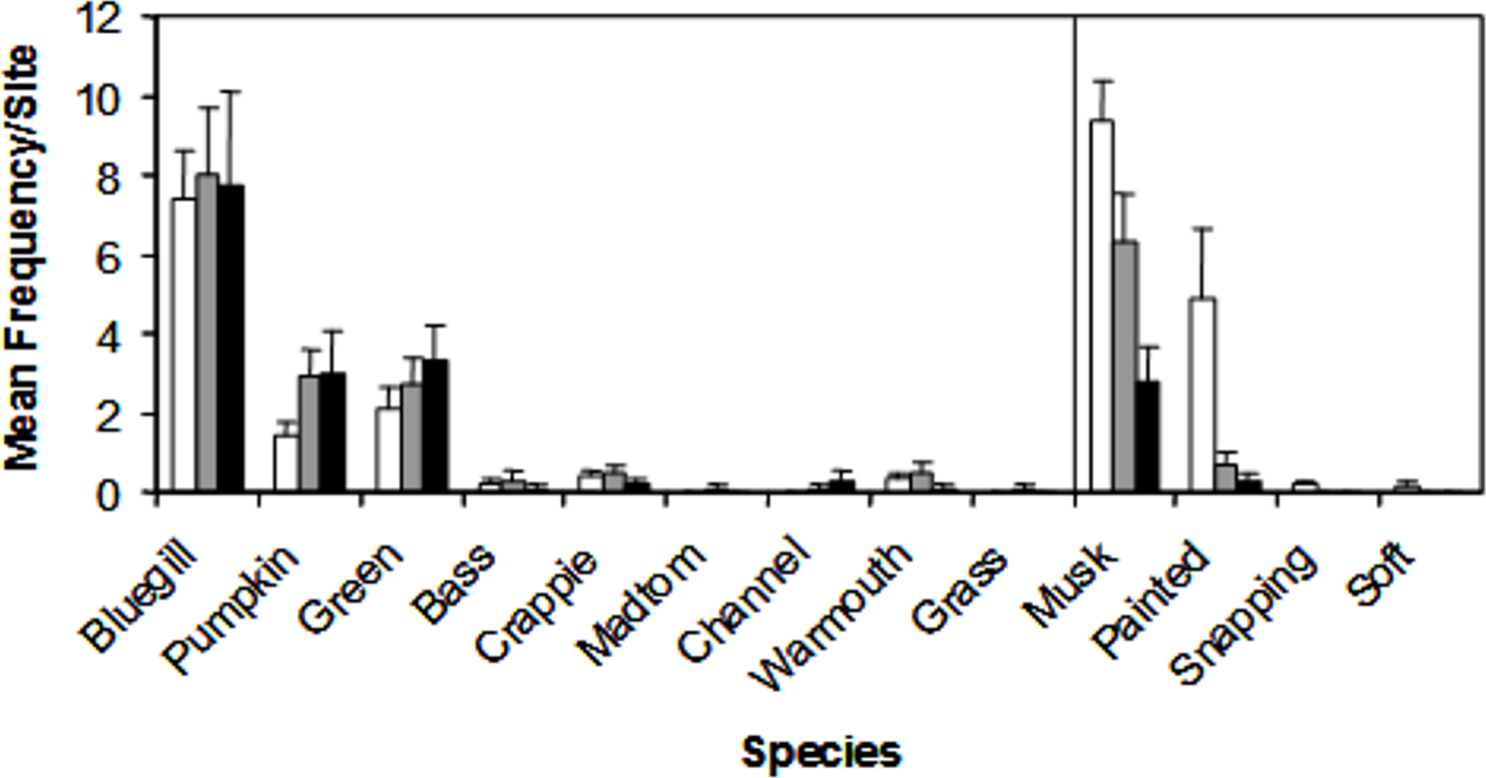
Mean catch frequency per site (+ SE) for each species encountered in the riverine (white bars), transitional (gray bars), and lacustrine zones (black bars) of Dow Lake. The abundance of turtles declined from the riverine zone to the lacustrine zone (i.e., along the depth/slope/turbidity gradient).

We encountered nine species of fish (bluegill, *Lepomis macrochirus*; pumpkinseed, *Lepomis gibbosus*; green sunfish, *Lepomis cyanellus*; largemouth bass, *Micropterus salmoides*; white crappie, *Pomoxis annularis*; brindled madtom, *Noturis miuris*; channel catfish, *Ictalurus punctatus*; warmouth sunfish, *Lepomis gulosus*; grass carp, *Ctenopharyngodon idella*) and four species of turtle (musk, *Sternotherus odoratus*; painted, *Chrysemys picta*; common snapping, *Chelydra serpentina*; spiny softshell, *Apalone spinifera*) during trapping. Of these species, three *Lepomis* species (bluegill, pumpkinseed, green) and two turtle species (musk and painted) represented >90% of the catch (Fig. 3), but all species were encountered in at least two sites during the study, and were therefore all included in the NMDS ordination.

### NMDS ordination

**Figure 4:**
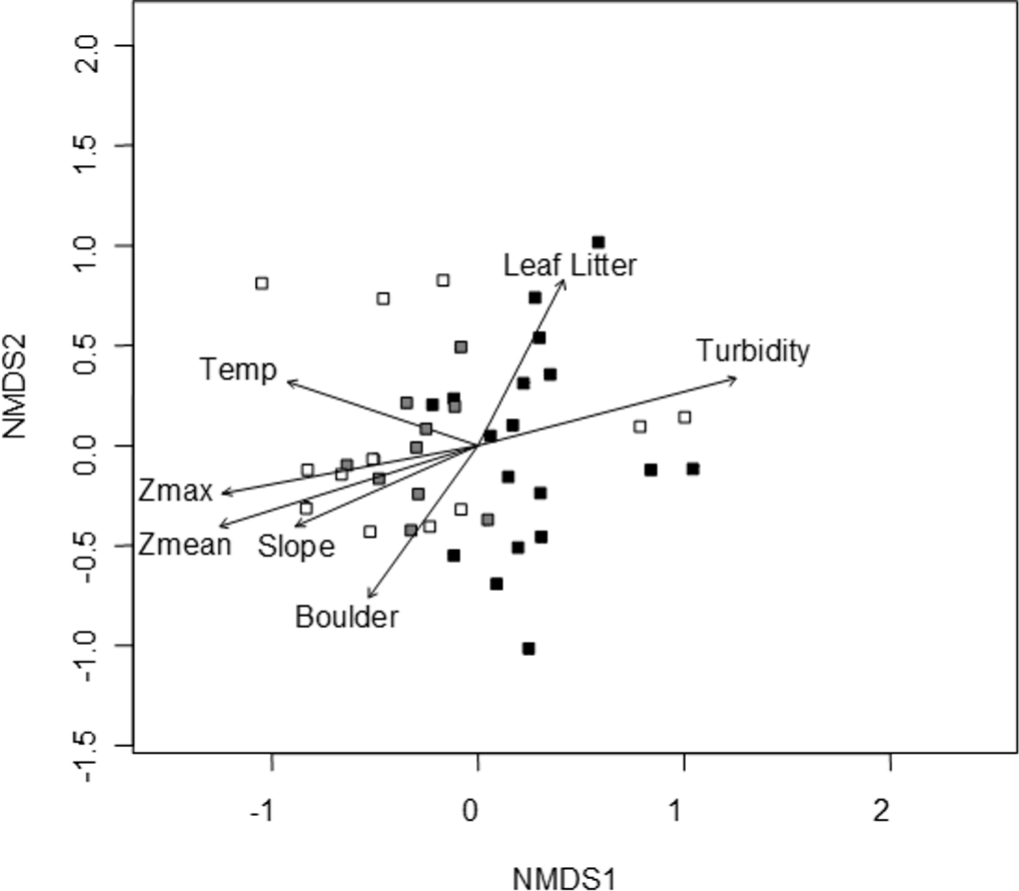
NMDS ordination displaying sites and significant environmental vectors. Site symbols are coded to represent their location within the reservoir: closed black boxes indicate sites in the riverine zone; closed gray boxes are transitional zone sites; and open boxes are lacustrine zone sites. The open boxes on the right side of the figure correspond to the sites indicated in Fig. 1 as “Riverine?,” otherwise there is little overlap between riverine and lacustrine sites. See Table 1 for environmental descriptive and fitting statistics.

**Figure 5:**
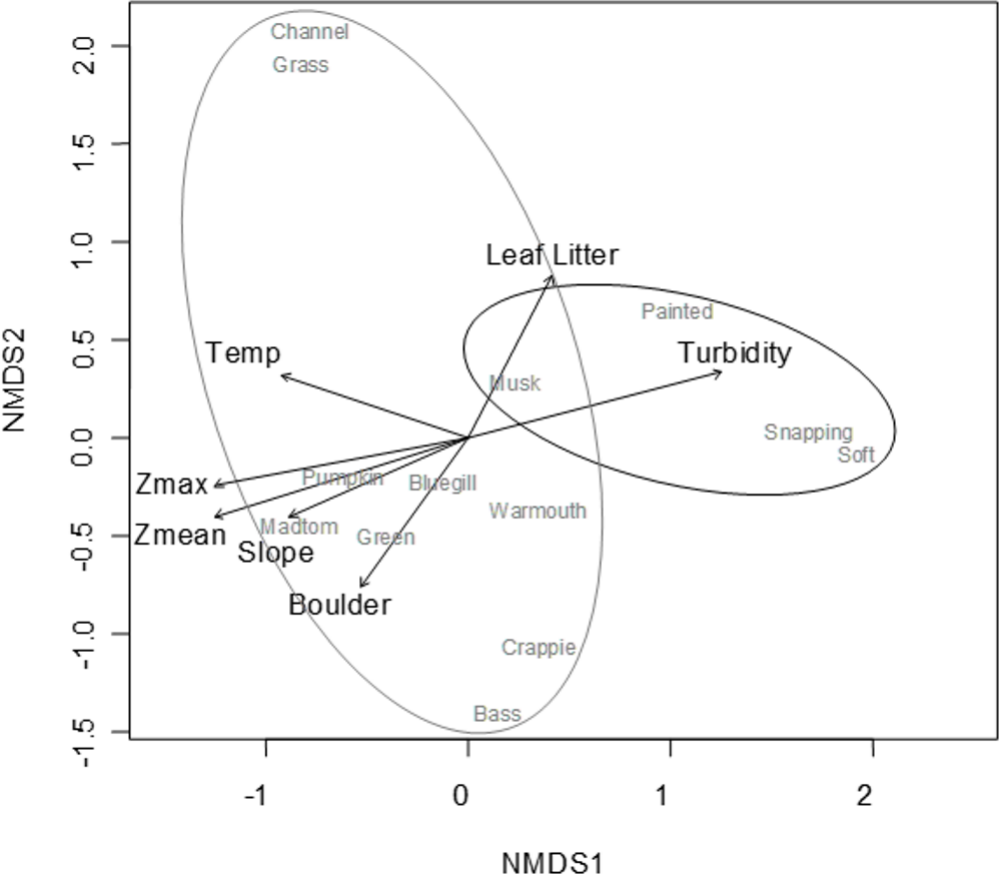
NMDS ordination displaying species objects and significant environmental vectors. The black ellipsoid highlights turtle objects, while the gray ellipsoid surrounds fish objects. See Table 1 for environmental descriptive and fitting statistics.

A convergent (stable) NMDS ordination was identified after 18 iterations with a stress of 18.94 (Figs. 4 and 5). Because the stress of an NMDS ordination will vary depending on the starting point (first iteration; Minchin, 1987), the analysis was run a number of times in order to ensure that the metaMDS function was performing well with the final stress falling between 18 and 20 in all cases. The analysis presented in Figs. 4 and 5 is the ordination that produced the lowest stress (18.94). As distance between site (Fig. 4) or species (Fig. 5) objects increases in the ordination plot, the similarity between them decreases. Descriptive and fitting statistics for environmental variables are provided in Table 1. The angle of a plotted vector (the best fit of the environmental variable) from the origin in Figs. 4 and 5 indicates the direction in which values are increasing, while the length of the vector represents its predictive power (the longer the vector, the higher its *R*^2^ value).

Sites in Fig. 4 are categorized according to the reservoir zonation scheme outlined in the introduction (Kimmel, Lind & Paulson, 1990). Note that two “lacustrine” sites (open boxes) are strongly associated with the cloud of “riverine” sites (black closed boxes), but that otherwise there is little overlap between the riverine and lacustrine sites (Fig. 4). This zonation structure can also be inferred by noting the direction of the Zmax and Zmean vectors relative to the location of sites. Littoral zone slope (depth in cm 3 m from shore) was positively correlated with the other depth measurements and turbidity was negatively correlated with depth (as in Fig. 2). The amount of leaf litter and large rocks (“boulder”) were both significant vectors and were negatively correlated with one another, but the direction of the gradient was different from that of the depth/slope/turbidity gradient. Similarly, temperature appears to represent a third distinct environmental gradient structuring the community.

In Fig. 5, site objects have been removed and species objects are displayed. There is a clear difference in the location of fish and turtle species, indicating that some sites in the ordination (Fig. 4) are characterized by high turtle catch frequency relative to fish, while other sites are characterized by high fish catch frequency relative to turtles. In order to better visualize this dynamic, fish and turtle catch frequencies (rather than rank-order observed abundance used in the ordination) were plotted against the best predictor variable for each of the three different environmental gradients identified in the NMDS analysis (zMean, Boulder, and Temperature; Table 1).

**Figure 6:**
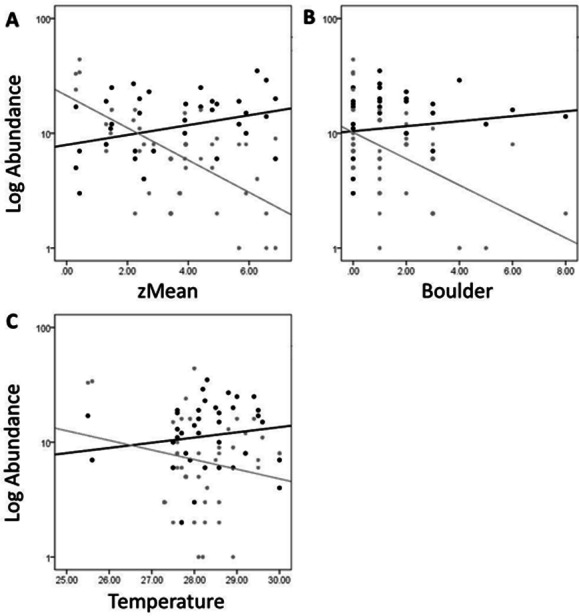
Log abundance of fish (black) and turtles (gray) for each site sampled plotted against the zMean (A), Boulder (B), and Temperature (C) environmental variables. The *R*^2^ for the trend lines can be found in the text of the results section.

**Figure 7:**
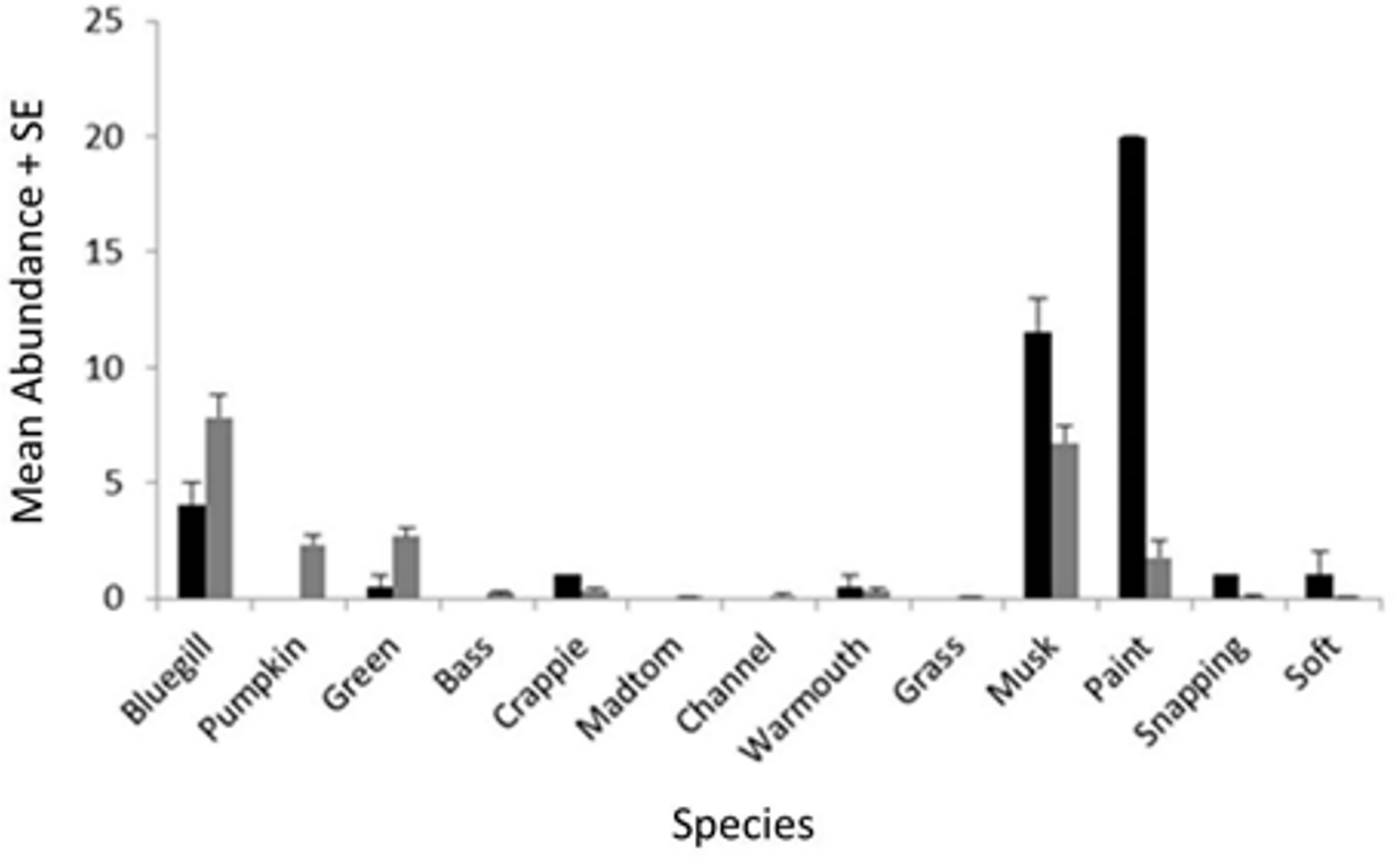
Mean abundance + standard error for each species in the NMDS ordination for cold (black) and warm (gray) sites (Fig. 6C).

When zMean was plotted against the log-catch frequency of fish and turtles (Fig. 6A), a simple linear regression showed that 45% of the variation was explained for turtles, but only 8% of variation for fish. Similarly, a regression of Boulder against log catch-frequency (Fig. 6B) explained 25% of the variation for turtles, but less than 2% for fish. Despite being a significant vector in the NMDS ordination, temperature had little explanative power for overall fish or turtle catch frequency (Fig. 6C). However, two sites had distinctly lower temperatures than the rest of the sites (Fig. 6C; Fig. 1 “cold”). These two cold sites were characterized by a much lower observed abundance of fish and a much higher (and diverse) observed abundance of turtles relative to the rest of the reservoir (Fig. 7) and is probably the dynamic most responsible for temperature being a significant vector in the NMDS ordination (Figs. 4 and 5).

## Discussion

Characterization of the littoral vertebrate community using NMDS ordination revealed that fish and turtles were broadly separated in ordination space and three distinctly different environmental gradients were significantly associated with the underlying observed abundances (Figs. 4 and 5). Observed turtle abundance was explained well by both the depth/turbidity gradient and the leaf-litter/boulder gradients independently, but neither of these gradients had much independent predictive power for observed fish abundance (Figs. 6A and 6B). Temperature was a poor predictor of observed abundance for both fish and turtles independently (Fig. 6C), though a plot of two anomalously cold sites against the rest of the reservoir revealed that these sites may be characterized by a lower observed fish abundance and higher observed turtle abundance (Fig. 7). Taken as a whole, these results suggest that the predictor (environmental) variables we used were appropriate for investigating turtle ecology in reservoirs, but that observed fish abundance is mediated by factors that were not modeled in the NMDS ordination. The variables from Table 1 that were most closely associated with the direction of spread for fish species objects in Fig. 5 (vectors not shown) were Chlorophyll and Woody Debris; these variables may be a good starting point for developing new environmental variables such as “distance to woody debris” or “plankton density”.

Despite being able to resolve a number of environmental variables that were significantly correlated with the observed abundance of fish and turtles, this result needs to be interpreted with caution. The decision to use traps was made primarily because of bias in the sampling efficiency of other methods for sampling the littoral zone for fish (Lapointe, Corkum & Mandrak, 2006), because methods such as electroshocking or seining would not allow investigation of the turtle community, but also because of logistical and financial constraints. That is, trapping was the best single method for concurrently sampling fish and turtles in the littoral zone of a reservoir available to us, but trapping is not without its own limitations (Fago, 1998).

First, there is an explicit assumption that a given individual is as likely to enter a trap in one location as in another location (trapping efficiency is assumed to be independent of site). While this seems a straightforward assumption, given that turtles were found more commonly at some sites than other sites, the presence of turtles in a trap may have dissuaded fish from entering. The same is equally true for small turtles entering a trap containing large fish. Second, in the case of this study, there are abundant species such as spot-fin shiners (*Notropis spilopterus*) present in the reservoir that are under-represented in traps. Similarly, both the largest and smallest individuals of a species are excluded; an observation that is of particular concern for larger turtle species such as snapping turtles because turtle populations are typically biased toward larger individuals (Ernst, Lovich & Barbour, 1994). Size and species biases, which apply to both fish and turtles, are the result of a combination of factors including mesh size, funnel size, bait type, and trap location. Lastly, sampling took place only during July and August, which means that we cannot comment on the spatial distribution of turtles or fish relative to reservoir environmental gradients at other times, which may well vary. However, the staggered (blocked) sampling design we employed may have actually worked in our favor because sampling is not temporally compressed (as in methods like electroshocking); spatial autocorrelation between adjacent sites is reduced by increasing temporal variability and using a blocked (rather than random) sampling design (Fortin & Dale, 2005), lending credence to our results. That is, any similarities in community composition between adjacent sites at different times should be more robust than if all the sites were sampled at the same time.

For turtles, the observed preference for turbid, shallow, and un-stratified areas may be simply in response to an increase in suitable habitat (Ruhl, 2013); the bottom of the entire riverine zone is easily accessible to turtles for foraging, which may in turn increase the abundance of turtles found along the edge of the reservoir where traps were placed. Another possibility is that because turtles and fish are both opportunistic generalist foragers (Aresco & James, 2005), there may be competitive displacement between fish and turtles. In the shallower areas turtles may be the superior littoral competitors, while in the deeper areas of the reservoir, fish may be the superior littoral competitor. This adaptation-based hypothesis (the compression hypothesis; Schoener, 1974) has been used to explain fish assemblage patterns (Werner & Hall, 1979) and may be driven by turbidity and predation efficiency (Vineyard & O’Brien, 1976; Abrahams & Kattenfeld, 1997) for fish. Turtle assemblages have been similarly examined in the context of resource partitioning (Luiselli, 2008), but to our knowledge have not been evaluated in the context of the compression hypothesis for lentic systems.

In addition to the depth/slope/turbidity gradient, a gradient of benthic resources (boulder/leaf-litter) was also significantly associated with community composition (Fig. 5), but seemed to be primarily driven by observed turtle abundance (Fig. 6B). On the surface, this observation suggests that fish and turtles are making disparate use of benthic resources in the littoral zone (as was seen in fish in Weaver, Magnuson & Clayton, 1997). Resource partitioning is known to occur in both aquatic turtles (Luiselli, 2008) and fish (Helfman, Collette & Facey, 1997), but to our knowledge, there have not been any studies that have investigated the potential for resource partitioning between fish and turtles. It is likely that there is some level of competition between fish and turtles for resources (e.g., for gastropod prey; Covich, 2010). If competition exists in a general sense among fish and aquatic turtle species, it is probably mediated by ontogeny due to shifts in diet and habitat use with size. Similarly, predation interactions would be mediated by ontogeny because fish populations are typically biased toward smaller individuals (Helfman, Collette & Facey, 1997) while turtles are biased toward larger individuals (Ernst, Lovich & Barbour, 1994). That is, large (rare) fish can eat juvenile turtles (Ernst, Lovich & Barbour, 1994), but usually the predation vector is observed as larger (common) turtles feeding on fish (Vogt & Guzman, 1988). Because our methods excluded the largest and smallest individuals (both fish and turtles), the impact of the benthic resource gradient on observed community composition needs to be interpreted with caution. While resource partitioning is a possibility, it is likely that the individuals that we encountered during our study are not normally predators of one another, so we may have inadvertently sub-sampled the proportion of the fish and turtle populations that are most likely to demonstrate resource-partitioning-like effects.

Alternatively, the leaf-litter/boulder variables may be pseudo-correlated with the depth/slope/turbidity gradient. That is, the inverse relationship between leaf-litter and boulder may be due to differences in wave-induced mixing between sites combined with the settling of suspended material along the horizontal axis of the reservoir. Sites that are exposed to waves on a regular basis have less fine and decomposing material due to scour relative to sites protected from waves (Severson, Nawrot & Eichholz, 2009); but because turbidity declines along the long-axis of the reservoir, riverine zone sites highly susceptible to waves may still have high proportions of fine material (Thornton, 1990a). Similarly, because the riverine zone receives a high amount of allochthonous material relative to the lacustrine zone (Thornton, 1990a), riverine sites may have higher proportions of leaf-litter. Therefore, the same ecological considerations used to explain shifts in observed turtle abundance according to turbidity and depth/slope in a reservoir may be at work here.

The third significant environmental gradient in the NMDS ordination was temperature. Unlike the other two gradients discussed above, neither observed fish nor turtle abundance was independently associated with temperature. Instead, a combination of low observed fish abundance and high observed turtle abundance at two particularly cold sampling sites relative to the rest of the reservoir caused this variable to be fitted significantly to the ordination. Both of the cold sites were in the upper-most portion of the riverine zone and were probably colder due to being more heavily influenced by lotic inputs.

That turtles would be captured more frequently in colder locations in a reservoir is at first counterintuitive, but the presence of cold (and oxic) water in close proximity to warm water in a reservoir may provide turtles with a thermoregulatory mechanism not as readily available in the littoral zone of a natural lake: cold lotic water meeting warm lentic water. This hypothesis is supported by studies of the most common turtle species we encountered in this study, which suggest these species thermoregulate via differential habitat use (musk turtles: Picard, Carriere & Blouin-Demers, 2011; painted turtles: Edwards & Blouin-Demers, 2007). If such is the case, it may be that turtles are congregating near colder areas in the riverine zone, which may in turn drive down fish abundance through either increased predation or competition. This may be a fertile area for future research of aquatic turtle ecology in reservoirs and their role in shaping the aquatic community.

In Figs. 1, 3 and 4, sites were classified by reservoir zone based largely on distance from inflowing water and the depth of stable stratification. This classification was useful as a tool to understand community differences between fish and turtles in this study (Figs. 3 and 4). However, two sites classified as lacustrine (“Riverine?” in Fig. 1 and open boxes on the right side of Fig. 4) were much more similar to riverine sites than lacustrine sites with respect to the fish and turtle community. This observation highlights the need for further research on the demarcation between reservoir zones in studies of fish and turtles. Both of these anomalous sites were positioned in relatively shallow embayments at the deep end of the reservoir whereas all other lacustrine sites were positioned on the shoreline immediately adjacent to deep water. Large embayments are known to exhibit conditions disparate to adjacent main-channel areas (Kimmel, Lind & Paulson, 1990), but the difference was surprising in this case given that each embayment was small and fed by 1st order ephemeral streams. While neither of these sites would be much affected by the turbid conditions like those found in the true high-flow riverine zone (due to their small watersheds), the depth profile and substrate composition may be similar enough to cause a shift in littoral species composition. This suggests that, for littoral habitats, it may be most appropriate to establish reservoir zones based on factors related to bathymetry, lotic flow, and stratification in both the main-channel and embayments rather than basing the zonation scheme on limnetic conditions along the long-axis of a reservoir (as in Kimmel, Lind & Paulson, 1990).

In summary, the sampled littoral vertebrate community of Dow Lake, particularly the turtles, was found to exhibit marked spatial differences in observed abundance along three environmental/resource gradients. The use of the reservoir zonation scheme for comparing turtle abundances between different areas of a reservoir appears to work well (as in Ruhl, 2013), but may not be appropriate for fish as currently defined. While the results presented here suggest that the vertebrate community of a reservoir may be spatially dynamic in a predictable way during July and August, further studies need to be conducted to (1) address trapping biases by utilizing multiple sampling methods, (2) incorporate ontogeny (e.g., temporally mediated shifts in resource-partitioning), (3) integrate individual preferences (e.g., migration of turtles), and (4) utilize additional predictor variables for fish. Lastly, there is a need for additional studies to explicitly investigate ecological interactions between aquatic turtles and fish in habitats where these organisms are sympatric and in high abundance, such as in the shallow areas of mid-latitude reservoirs.

## Additional Information and Declarations

### Field Study Permissions

The following information was supplied relating to field study approvals (i.e., approving body and any reference numbers): Ohio Department of Natural Resources #464 sampling permit.
